# Enhanced and Stable Upconverted White-light Emission in Ho^3+^/Yb^3+^/Tm^3+^-doped LiNbO_3_ Single Crystal via Mg^2+^ Ion Doping

**DOI:** 10.1038/s41598-017-15367-9

**Published:** 2017-11-07

**Authors:** Lili Xing, Weiqi Yang, Jincheng Lin, Mei Huang, Yuqi Xue

**Affiliations:** 1 Sino-French Institute of Nuclear Engineering and Technology, Sun Yat-sen University, Zhuhai, 519082 P. R. China; 2 Zhuhai Campus Experimental Teaching Center, Sun Yat-sen University, Zhuhai, 519082 P. R. China

## Abstract

A strategy to enhance the upconversion white-light intensity via Mg^2+^ ion doping was demonstrated in Ho^3+^/Yb^3+^/Tm^3+^/LiNbO_3_ single crystal. It is found Mg^2+^ ion doping affects the crystal field symmetry around RE^3+^ ions and enhance the upconversion emission intensity. Bright white-light is obtained when the Mg^2+^ ion concentration is 0.5 mol% in the melt. And the CIE coordinates are hardly changed with Mg^2+^ ion doping. In addition, the upconversion mechanism is discussed in detail. It is observed the longer lifetimes of intermediate levels result in the lower upconversion photon numbers, which are beneficial to the upconversion process. Therefore, Mg^2+^ ion doped Ho^3+^/Yb^3+^/Tm^3+^/LiNbO_3_ single crystals would have potential applications in stable white-light devices and photoelectric instruments.

## Introduction

Recently, trivalent rare earths (RE^3+^) doped upconverting white-light materials have attracted great attentions due to their potential applications in color display, bio-label, solar cell, optical temperature sensor, and so on^1–5^. As known, upconversion is an anti-stokes process which can convert two or more low-energy photons into a high-energy photon. To avoid the intrinsic color balance, device complication and high cost problems when using multiphosphors, the blue, green and red emissions are expected to be generated simultaneously in a host material. The single white-light phosphors are pursued^6–8^. At present, many researches focus on the tri-doped Yb^3+^, Ho^3+^ and Tm^3+^ system in different host materials^9–11^, where the Yb^3+^ ions act as sensitizers to efficiently absorb the pump photons and transfer their energy to Tm^3+^ or Ho^3+^ ions. As a result, the blue emission (Tm^3+^), green and red emissions (Ho^3+^) are achieved, which can further produce the white-light emission.

Since the upconversion emission intensity and chromaticity play key roles in practical applications for white-light materials, it is of technological and scientific importance to look for an effective way to enhance the upconversion emission intensity and obtain a stable white-light emission. During the past few years, in order to improve the properties of upconversion white-light materials, much attentions have been focus on the modulation of RE^3+^ ion concentration, controlled synthesis of host material, suitable selection of excitation source, and the optimization for temperature and some other affecting factors, etc^12–15^. However, the obtained results are not satisfying so far because of their relevant limitations. Generally, high RE ion concentrations are required to guarantee the upconversion emission intensity but detrimental for the crystal quality of host material, too high RE^3+^ ion concentrations may induce the quenching of upconversion luminescence. And meanwhile, the adjustment of host material or affecting factors may result in the application difficulty and operation complexity.

Worthy of notice, in addition to the presence of RE^3+^ ions in upconverting white-light materials, the codoping of various non-luminous ions may cause the improvement of luminescence behavior by modifying the local environment around the emitters^16^. Recently, a few reports have focused on the enhancement of upconversion emission by codoping non-lanthanides (Li^+^, Mg^2+^, Na^+^, Sc^3+^ etc.) in RE^3+^ ions doped upconverting materials^17–19^. Luitel *et al*. have studied the effects of M^+^ (M = Li, Na, K, Rb) ion in CaMoO_4_:RE^3+^,Yb^3+^ (RE = Er, Ho, Tm) phosphors^20^. Guo *et al*. revealed that the Li^+^ doping could enhance the emission intensity of Yb^3+^/Ho^3+^ codoped Lu_6_O_5_F_8_ nanoparticles^21^. Many researches show that the codoping of non-luminous ions into RE^3+^ ions doped upconverting materials is a promising way to increase the intensity of upconversion emission. But the relative studies are still limited, to the best of our knowledge, few reports concerned about the influence of non-luminous ions on upconversion white-light properties in RE^3+^ ions doped materials to date.

In this article, LiNbO_3_ single crystal was used as host material, its lower phonon energy guarantees the higher upconversion efficiency. Mg^2+^ ion was introduced into Ho^3+^/Yb^3+^/Tm^3+^ tri-doped LiNbO_3_ single crystals due to its small ionic radius. Here, we represent a new strategy to improve the properties of upconversion white-light emission. Under 980 nm excitation, the influences of Mg^2+^ ion on the intensity and color tunability of upconversion white-light emission were demonstrated and the rational explanation was given. Preferably, the multi-function of LiNbO_3_ single crystal will create sufficient conditions for opening up new perspectives to the studies of integration and tiny devices.

## Results and Discussion

**Figure 1:**
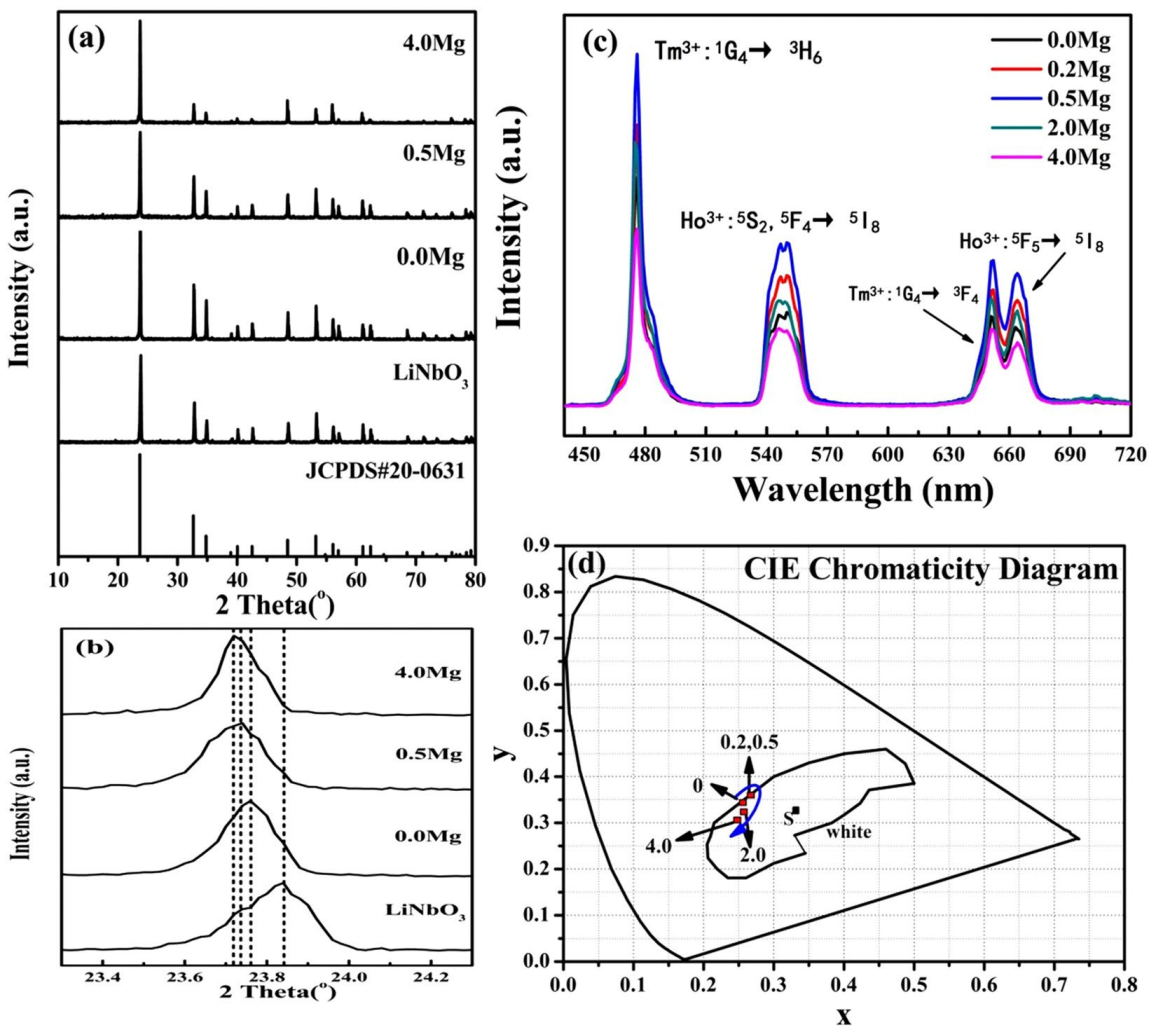
(**a**) XRD patterns of pure LiNbO_3_ and Ho^3+^/Yb^3+^/Tm^3+^/LiNbO_3_ single crystals doped with different Mg^2+^ ion concentrations. (**b**) Amplified the main diffraction peaks of pure LiNbO_3_ and Ho^3+^/Yb^3+^/Tm^3+^/LiNbO_3_ single crystals doped with different Mg^2+^ ion concentrations. (**c**) Upconversion emission spectra of Ho^3+^/Yb^3+^/Tm^3+^/LiNbO_3_ single crystals doped with different Mg^2+^ ion concentrations under 980 nm excitation at room temperature. (**d**) Calculated color coordinates (x, y) of the upconversion emissions for Ho^3+^/Yb^3+^/Tm^3+^/LiNbO_3_ single crystals doped with different Mg^2+^ ion concentrations under 980 nm excitation. The S point is the standard white-light coordinate.

Table 1 presents the molar compositions of cations in the melt or crystal for Mg^2+^ doped Ho^3+^/Yb^3+^/Tm^3+^/LiNbO_3_ crystals. It can be seen that the Mg^2+^ and RE^3+^ ions are introduced into the crystals successfully. With increasing Mg^2+^ ion concentrations in the melt, Mg^2+^ ion concentrations in the crystals are increased evidently. However, the total concentrations of RE^3+^ ions in the crystals are decreased slightly with increasing Mg^2+^ ion concentrations, which could be considered as unchanged. In addition, Fig. 1(a) shows the powder XRD patterns of pure LiNbO_3_ and Ho^3+^/Yb^3+^/Tm^3+^/LiNbO_3_ single crystals doped with different Mg^2+^ ion concentrations. As shown, all the diffraction peaks of the samples can be well indexed to the standard LiNbO_3_ phase (JCPDS file no. 20-0631), no secondary phases were identified. It can be concluded that the doping ions do not alter the phase structure of host material, and the ionic radius differences between doping ions and host ions result in the variations of diffraction peak intensities. To further investigate the effect of Mg^2+^ ions on the structure of Ho^3+^/Yb^3+^/Tm^3+^/LiNbO_3_ single crystal, the main diffraction peak is amplified, as shown in Fig. 1(b). In general, the RE^3+^ ions with larger ionic radius (r_Ho_^3+^ = 0.89 Å, r_Yb_^3+^ = 0.86 Å, r_Tm_^3+^ = 0.87 Å, r_Li_^+^ = 0.68 Å, r_Nb_^5+^ = 0.69 Å) will enter into the LiNbO_3_ crystals in the form of lattice substitution. So the host lattice is expanding, which could lead to the shift of the main diffraction peak towards smaller angle, as shown in Fig. 1(b). However, Mg^2+^ ions with smaller ionic radius (r_Mg_^2+^ = 0.66 Å) may enter into the crystals in the form of lattice substitution or interstitial. For lattice substitution, the host lattice shrinking is induced since the ionic radius of Mg^2+^ ion is smaller than that of Li^+^ or Nb^5+^ ions, corresponding to the shift of the main diffraction peak towards larger angle. By contrast, for the interstitial, the host lattice expanding occurs, resulting in the shift of the main diffraction peak towards smaller angle. Based on the above mentioned, the site occupancy of Mg^2+^ ion is mainly indentified by the shift of the main diffraction peak. It can be seen from Fig. 1(b) that with increasing Mg^2+^ ion concentrations, the main diffraction peak shifts gradually towards smaller angles, which means the lattice is expanding and the Mg^2+^ ions enter into the crystals in the form of interstitial. As a result, the occupation of interstitial site for Mg^2+^ ion can tailor the local crystal field around RE^3+^ ions in the host lattice, which will affect its luminescence properties.

**Table 1:** Molar compositions of cations in the melt or crystal for Ho^3+^/Yb^3+^/Tm^3+^/LiNbO_3_ crystals with various Mg^2+^ ions concentrations.

Samples	CMg^2+^ in melt (mol%)	CMg^2+^ in crystal (mol%)	CRE^3+^ in melt (mol%)			CRE^3+^ in crystal (mol%)		
			Ho^3+^	Yb^3+^	Tm^3+^	Ho^3+^	Yb^3+^	Tm^3+^
0.0 Mg	0	0	0.025	2.0	0.2	0.016	3.04	0.298
0.2 Mg	0.2	0.28	0.025	2.0	0.2	0.019	3.03	0.296
0.5 Mg	0.5	0.66	0.025	2.0	0.2	0.021	3.03	0.294
2.0 Mg	2.0	2.30	0.025	2.0	0.2	0.024	3.01	0.293
4.0 Mg	4.0	4.04	0.025	2.0	0.2	0.025	3.01	0.291

The upconversion emission spectra of Ho^3+^/Yb^3+^/Tm^3+^/LiNbO_3_ single crystals doped with various Mg^2+^ ion concentrations under 980 nm excitation are shown in Fig. 1(c). As shown, the blue emission has a luminescence peak at 477 nm that corresponds to Tm^3+^: ^1^G_4_ → ^3^H_6_ transition; the green emission band centered around 549 nm is contributed to Ho^3+^: ^5^S_2_, ^5^F_4_ → ^5^I_8_ transition; and the red emission has luminescence peaks at 652 nm and 665 nm originating from Tm^3+^: ^1^G_4_ → ^3^F_4_ transition and Ho^3+^: ^5^F_5_ → ^5^I_8_ transition, respectively. From Fig. 1(c), it is observed that the intensities of blue, green and red emissions increase first and then decrease with increasing the Mg^2+^ ion concentrations. The optimum Mg^2+^ ion concentration is 0.5 mol% in the melt, corresponding to 0.66 mol% in the single crystal. In our case, we argue that Mg^2+^ ion with small ionic radius can be doped into the host lattice easily in the form of interstitial according to the XRD results, and this will break the symmetry of the crystal field around the rare earth ions. If the rare earth ions are placed at a low symmetry site, the forbidden transitions will be weakened, leading to the enhancement of upconversion emission. The similar phenomena are also found in the research reports, such as refs^22,23^. But when the Mg^2+^ ion concentration is above optimum concentration, the doped ions may cause the lattice distortion around the rare earth ions, resulting in the quenching of the upconversion emission. To investigate the color tunability, Fig. 1(d) shows the CIE coordinates of Ho^3+^/Yb^3+^/Tm^3+^/LiNbO_3_ single crystals doped with various Mg^2+^ ion concentrations. It can be seen that the CIE coordinates of samples undoped and doped Mg^2+^ ion are located in the white-light region basically. Moreover, the CIE coordinates have the trend of shift towards green/red region first and then tend to shift towards blue region with increasing the Mg^2+^ ion concentrations. But it should be noted that it shows little color tunability under Mg^2+^ ion doping. So the Mg^2+^ ion doped Ho^3+^/Yb^3+^/Tm^3+^/LiNbO_3_ single crystals may be suitable for making the non-tunable white-light display devices.

**Figure 2:**
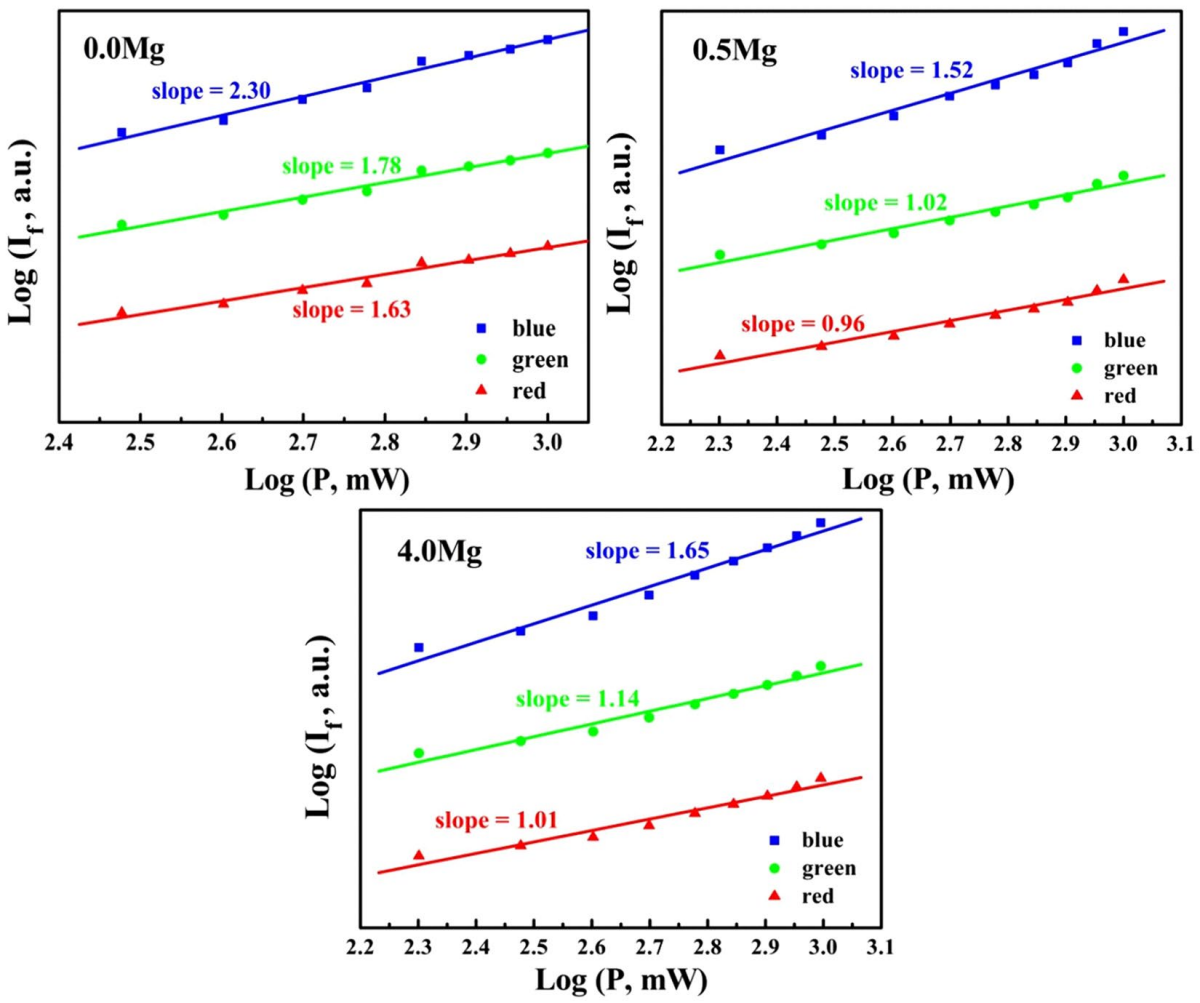
Dependences of upconversion emission intensities on excitation powers for Ho^3+^/Yb^3+^/Tm^3+^/LiNbO_3_ single crystals doped with different Mg^2+^ ion concentrations under 980 nm excitation.

**Figure 3:**
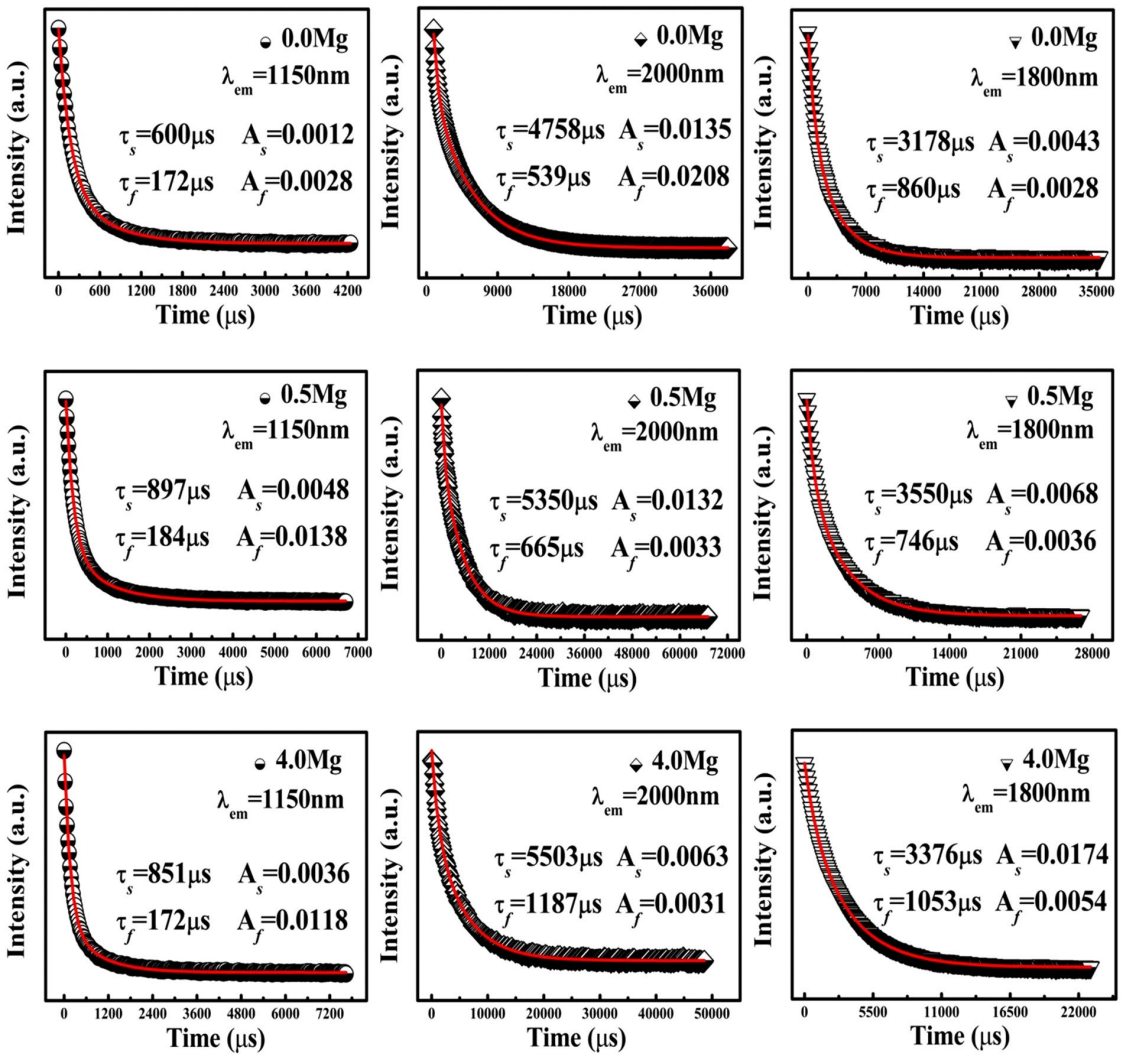
Upconversion luminescence decay dynamics of the ^5^I_6_ (λ_em_ = 1150 nm) and ^5^I_7_ (λ_em_ = 2000 nm) levels of Ho^3+^ ions as well as ^3^F_4_ (λ_em_ = 1800 nm) levels of Tm^3+^ ions in the Ho^3+^/Yb^3+^/Tm^3+^/LiNbO_3_ single crystals doped with different Mg^2+^ ion concentrations under 980 nm excitation.

To analyze the possible white-light upconversion mechanism in Mg^2+^ ions doped Ho^3+^/Yb^3+^/Tm^3+^/LiNbO_3_ single crystal, the dependences of upconversion emission intensities on pump powers are measured under 980 nm excitation, as shown in Fig. 2. Without Mg^2+^ ion doping, the slopes of blue, green, and red emissions for Ho^3+^/Yb^3+^/Tm^3+^/LiNbO_3_ single crystal are 2.30, 1.78, and 1.63, respectively. It can be obtained that the blue emission is a three-photon process, the green and red emission are two-photon processes. When the Mg^2+^ ion concentration in the melt is 0.5 mol%, the slopes of blue, green, and red emissions are 1.52, 1.02, and 0.96, respectively. And when the Mg^2+^ ion concentration in the melt reaches up to 4.0 mol%, the above values are 1.65, 1.14, and 1.01, respectively. As known, the slopes deviating from the integer values (3 or 2 or 1) are attributed to the competition between the linear decay and the upconversion processes for the depletion of the intermediate excited states and the local thermal effect^24,25^. These results indicate that the blue emission is a two-photon process, the green and red emission are one-photon processes with Mg^2+^ ion doping. As a supplement, the luminescence decay behaviors of Ho^3+^: ^5^I_6_ → ^5^I_8_ (λ_em_ = 1150 nm), Ho^3+^: ^5^I_7_ → ^5^I_8_ (λ_em_ = 2000 nm), and Tm^3+^: ^3^F_4_ → ^3^H_6_ (λ_em_ = 1800 nm) are investigated, as shown in Fig. 3. It can be easily seen that all the emission intensities decay exponentially. The double-exponential is adopted to fit the experiment data using the equation (1)^26^.

1 $$I({\rm{t}})={I}_{0}+{A}_{s}{e}^{-t/{\tau }_{s}}+{A}_{f}{e}^{-t/{\tau }_{f}}$$

where *I*(*t*) is the fluorescence intensity at time *t*, *I*_0_ stands for the background fluorescence intensity, *τ*_*s*_ and *τ*_*f*_ represent slow fluorescence lifetime and fast fluorescence lifetime, *A*_*s*_ and *A*_*f*_ are the weight factor of slow fluorescence lifetime and fast fluorescence lifetime, respectively. The lifetime of fluorescence level *τ* can be calculated according to the fitting results by equation (2).

2 $$\tau =\frac{{A}_{{\rm{s}}}{{\tau }_{s}}^{2}+{A}_{f}{{\tau }_{f}}^{2}}{{A}_{{\rm{s}}}{\tau }_{s}+{A}_{f}{\tau }_{f}}$$

The obtained lifetime values are shown in Table 2. It can be observed that the lifetime values of intermediate levels are increased with Mg^2+^ ion doping, resulting in the decrease of upconversion photon numbers. We believe that the longer lifetime of intermediate level is beneficial to the upconversion process, leading to the stronger upconversion emission intensity. But when the Mg^2+^ ion concentration in the melt is too high (4.0 mol% in this article), the lattice distortion plays an important role in upconversion process, so the upconversion luminescence is not emitted effectively though the lifetime of its intermediate level is long, the upconversion emission intensity is decreased consequently. Furthermore, the decay curve analysis of the excited levels involved in the following upconversion emission processes Tm^3+^: ^1^G_4_ → ^3^H_6_ (λ_em_ = 477 nm), Ho^3+^: ^5^F_4_, ^5^S_2_ → ^5^I_8_ (λ_em_ = 550 nm), and Ho^3+^: ^5^F_5_ → ^5^I_8_ (λ_em_ = 665 nm) are performed and calculated, the obtained lifetime values are shown in Table 1. It is suggested that the proper Mg^2+^ ion incorporation modifies the crystal field and results in the fast emitting of upconversion luminescence, and hence the upconversion emission intensity is enhanced. But the excessive Mg^2+^ ion concentration is detrimental to the enhancement of upconversion emission intensity.

**Table 2:** Lifetime values for ^5^I_6_ (λ_em_ = 1150 nm), ^5^I_7_ (λ_em_ = 2000 nm), ^5^F_5_ (λ_em_ = 665 nm), ^5^F_4_, ^5^S_2_ (λ_em_ = 550 nm) levels of Ho^3+^ ions as well as ^3^F_4_ (λ_em_ = 1800 nm), ^1^G_4_ (λ_em_ = 477 nm) levels of Tm^3+^ ions in the 0.025Ho^3+^/2.0Yb^3+^/0.2Tm^3+^/LiNbO_3_ single crystals doped with xMg^2+^ ions (x mol% = 0.0, 0.5, 4.0) under 980 nm excitation.

Samples	^5^I_6_ (ms) (λ_em_ = 1150 nm)	^5^I_7_ (ms) (λ_em_ = 2000 nm)	^3^F_4_ (ms) (λ_em_ = 1800 nm)	^1^G_4_ (μs) (λ_em_ = 477 nm)	^5^F_4_, ^5^S_2_ (μs) (λ_em_ = 550 nm)	^5^F_5_ (μs) (λem = 665 nm)
0.0Mg	0.43	4.20	2.80	209	127	262
0.5Mg	0.63	5.21	3.27	149	57	192
4.0Mg	0.58	5.09	3.17	235	145	266

**Figure 4:**
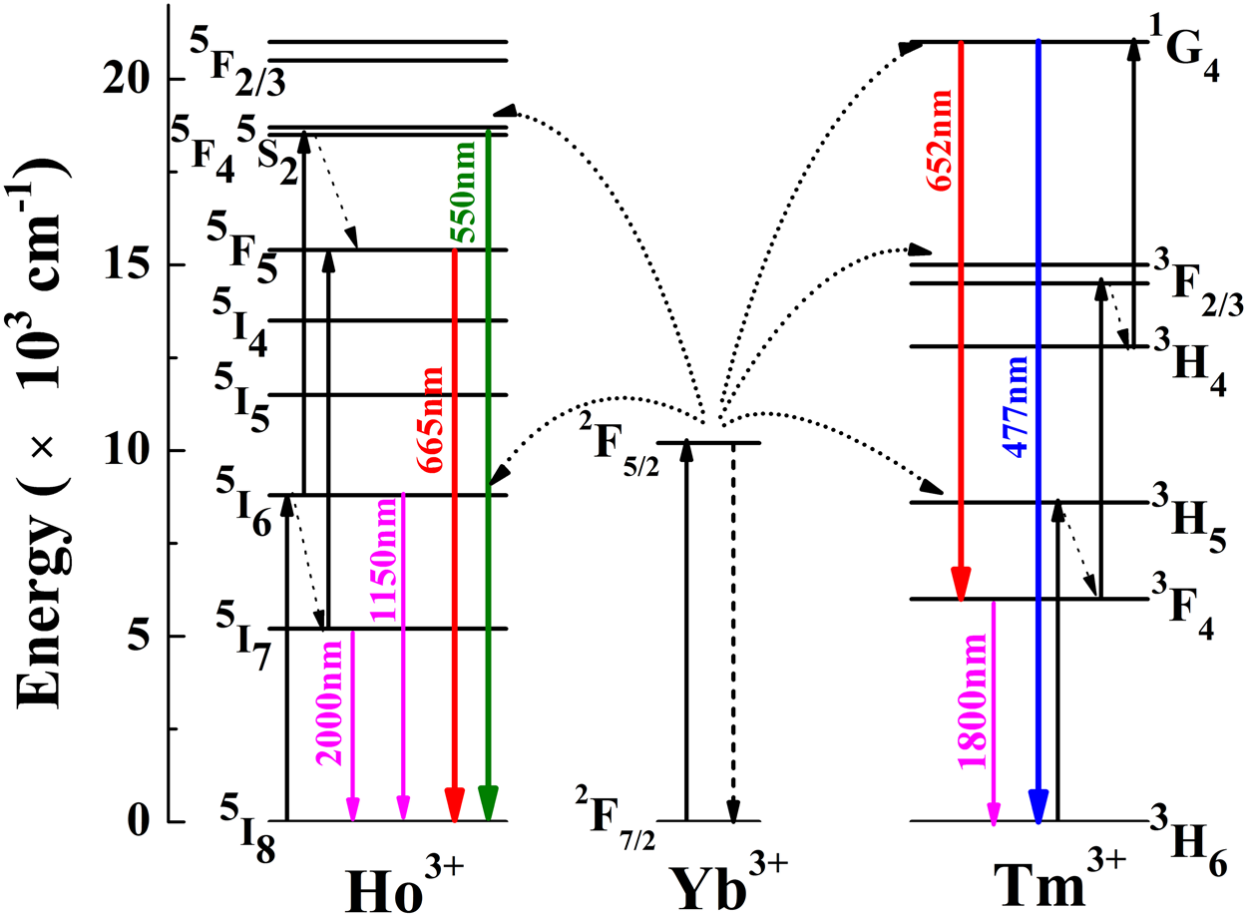
Schematics of the populating and upconversion luminescence processes for the blue, green and red emissions in the Mg^2+^ ions doped Ho^3+^/Yb^3+^/Tm^3+^/LiNbO_3_ system under 980 nm excitation.

The Schematics of populating and upconversion luminescence processes for the blue, green and red emissions in the Mg^2+^ ions doped Ho^3+^/Yb^3+^/Tm^3+^/LiNbO_3_ system under 980 nm excitation have been described in Fig. 4. From Fig. 4, it can be seen that Yb^3+^ ions act as sensitizers to absorb laser photons and transfer their energy to Ho^3+^ and Tm^3+^ ions effectively. Through two successive energy transfer processes from Yb^3+^ ions to Ho^3+^ ions, the ^5^F_4_, ^5^S_2_ levels and ^5^F_5_ levels of Ho^3+^ ions are populated, which generate the upconversion green and red emissions, respectively. Similarly, through three successive energy transfer processes from Yb^3+^ ions to Tm^3+^ ions, the upconversion blue emissions and weak red emissions are obtained originating from the ^1^G_4_ → ^3^H_6_ and ^1^G_4_ → ^3^F_4_ transitions of Tm^3+^ ions. In the upconversion processes, Mg^2+^ ion is not the luminescent ion. The doping Mg^2+^ ions can impact the lifetimes of excited levels, and further influence the upconversion emission intensity.

## Conclusion

Mg^2+^-doped Ho^3+^/Yb^3+^/Tm^3+^/LiNbO_3_ single crystals have been successfully prepared by Czochralski method. Bright upconversion white-light emission is achieved under 980 nm excitation at room temperature. It is found that Mg^2+^ and RE^3+^ ions could not alter the phase structure of host material, Mg^2+^ ions enter into the single crystals in the form of interstitial. The intensities of upconversion emissions are increased firstly and decreased subsequently with increasing Mg^2+^ ion concentrations. The optimum Mg^2+^ ion concentration is 0.5 mol% in the melt. The red, green and blue emissions in this system can be ascribed to Ho^3+^: ^5^F_5_ → ^5^I_8_, Tm^3+^: ^1^G_4_ → ^3^F_4_; Ho^3+^: ^4^S_2_, ^5^F_4_ → ^5^I_8_ and Tm^3+^: ^1^G_4_ → ^3^H_6_ transitions, respectively. The research results indicate that Mg^2+^ ion doping could not change the upconversion processes of Ho^3+^, Yb^3+^, and Tm^3+^ ions in LiNbO_3_ single crystals. The enhancement of upconversion emission intensity is mainly attributed to the increase of lifetimes for intermediate levels and the decrease of lifetimes for luminescent levels. Besides, it can be obtained that the CIE coordinates of upconversion emissions are almost unchanged with Mg^2+^ ion doping, which show ideal non-tunable white-light emissions. Such excellent white-light in Mg^2+^ ion doped Ho^3+^/Yb^3+^/Tm^3+^/LiNbO_3_ single crystals make it have potential applications in stable white-light devices and photoelectric instruments. This method would stimulate the further discovery to enhance the upconversion white-light intensity and fabricate other stable white-light materials.

## Methods

### Sample preparation

The Ho^3+^/Yb^3+^/Tm^3+^/LiNbO_3_ single crystals doped with different Mg^2+^ ion concentrations were grown by the Czochralski method. The concentrations of Ho^3+^, Yb^3+^ and Tm^3+^ ions in the melts were 0.025 mol%, 2.0 mol% and 0.2 mol%, respectively. And the Mg^2+^ ion concentrations in the melts are 0.0 mol%, 0.2 mol%, 0.5 mol%, 2.0 mol% and 4.0 mol%. The single crystals doped with different Mg^2+^ ion concentrations in the melt are denoted as 0.0 Mg, 0.2 Mg, 0.5 Mg, 2.0 Mg and 4.0 Mg in this article. The raw materials were Li_2_CO_3_, Nb_2_O_5_, Ho_2_O_3_, Yb_2_O_3_, Tm_2_O_3_ and MgO with 4 N purity. Firstly, the doped LiNbO_3_ polycrystals were prepared by high temperature solid state method. The raw materials were weighed and thoroughly mixed for 48 h, underwent a heat treatment of 750 °C for 2 h to resolve Li_2_CO_3_ into Li_2_O and CO_2_, and then sintered at 1150 °C for 10 h to form polycrystals. Secondly, the doped LiNbO_3_ single crystals were grown along the [0 0 1] direction using a diametercontrolled Czochralski apparatus. To grow crystals with good quality, the following optimum growth conditions were selected: the temperature gradient above the melt was 25 °C/mm, the pulling rate was 0.2 mm/h, and the seed rotation rate was 28 rpm. After growth, the crystals were cooled down to room temperature at a speed of 30 °C/h. For phase structure analyses, the samples were grinded into powder using an agate mortar. And for optical tests, Y-cut plates of the samples were cut and polished.

### Data availability statement

The datasets generated during and/or analysed during the current study are available from the corresponding author on reasonable request.

### Characterization

The Inductively Coupled Plasma Mass Spectrometry (ICP-MS) with Optima-7500 type was used to measure the mass fractions of Mg^2+^ ions and rare earth ions (RE^3+^) in the single crystals. To identify the crystallization phase, X-ray diffraction spectra of Mg^2+^ ion doped Ho^3+^/Yb^3+^/Tm^3+^/LiNbO_3_ single crystals were measured by an XRD-6000 diffractometer using a copper Kα radiation source. The upconversion luminescence spectra were recorded by Zolix-SBP300 grating spectrometer equipped with CR131 photomultiplier under 980 nm excitation. In the measurement of luminescence decay dynamics, the continuous wave from 980 nm laser diode was tuned into pulsing by a signal generator, and the luminescence decay curves were measured by a digital phosphor oscilloscope (Tektronix DPO 4140). The CIE chromaticity coordinate for the upconversion fluorescence of Mg^2+^-doped Ho^3+^/Yb^3+^/Tm^3+^/LiNbO_3_ single crystal was calculated based on the 1931 CIE standard and marked in the CIE standard chromaticity diagram.
